# Evidence on prevalence of caesarean sections and factors influencing uptake in Ghana: a scoping review

**DOI:** 10.1186/s12978-026-02317-w

**Published:** 2026-04-14

**Authors:** Stella Mahama, Louisa Lawrie, Jamini Dimri, Mairead Black

**Affiliations:** 1 Ghana Scholarship Secretariat, Accra, Ghana; 2 Ghana Health Service, Health Promotion Division, Accra, Ghana; 3 School of Medicine, Medical Sciences and Nutrition, Institute of Applied Health Sciences, University of Aberdeen, Aberdeen, Scotland UK; 4 Health Psychology Group, School of Medicine, Medical Sciences and Nutrition, Institute of Applied Health Sciences, University of Aberdeen, Aberdeen, Scotland UK; 5 Aberdeen Centre for Women’s Health Research, University of Aberdeen, Aberdeen, Scotland UK; 6 Aberdeen Maternity Hospital, Aberdeen, Scotland UK; 7 Aberdeen, Scotland, UK

**Keywords:** Caesarean sections, Rates, Factors, Optimal use, Barriers, Facilitators, Ghana

## Abstract

**Background:**

Ghana’s caesarean section (CS) rate has increased from 16% in 2017 to 21% in 2022 which matches the global CS rate (Ghana Demographic and Health Survey 2022) (Ghana Health Service 2016 Annual REport. 2017). Given that the World Health Organisation (WHO) recommends a range of 10% to 15% per live birth, Ghana, as of 2017, joined other countries that have exceeded this range (WHO Statement on Caesarean Section Rates. 2015) (Journal of Public Health (Germany) 25(5):557–64, 2017). This review set out to explore the prevalence of CS across regions of Ghana and to understand influences on CS uptake.

**Methods:**

The Joanna Briggs Institute methodological guidelines for scoping reviews were followed, and the review was reported in line with the Preferred Reporting Items for Systematic and Meta-Analyses extension for scoping reviews (PRISMA-ScR). Literature searches were conducted in MEDLINE, PsycINFO, EMBASE, Web of Science and CINAHL (EBSCO). The review included primary qualitative, quantitative, mixed methods studies, systematic reviews, scoping reviews, and grey literature. Narrative synthesis was used to present CS prevalence. The Theoretical Domains Framework (TDF) was used to assess factors influencing CS uptake.

**Results:**

Substantial variation in CS uptake is evident across Ghana regions, ranging from 15.7% (Menoufia Medical Journal 35, 2022) to 26% (BMC Pregnancy and Childbirth 19(1), 2019) in the Northern and 6.6% (BMC Pregnancy and Childbirth 18(1), 2018) to 40.6% (Texila International Journal of Public Health 11(2), 2023) across the Southern sector. Quantitative factors such as maternal age (older women), free maternal healthcare policy, and high household socio-economic status were among the specific factors influencing CS uptake in Ghana (Pan African Medical Journal 29, 2018), (BMC Pregnancy Childbirth 21(1), 2021), (BMC Pregnancy Childbirth 22(1), 2022), (Health Services Insights 2024), (Glob J Health Sci 6(4):9–21, 2014). Despite this, and several reports on clinical and demographic factors associated with CS uptake, there is very little in-depth evidence published on the nature of the barriers and facilitators to CS uptake; thus, future qualitative research is warranted.

**Conclusion:**

The review found variation in CS prevalence across regions of Ghana, ranging from 6.6% to 40.6%. The reason for this variation is not well understood. The limited evidence demonstrating potential influences on CS uptake in Ghana is mainly quantitative. Hence, we recommend further qualitative research with a range of stakeholders to better understand in-depth contextual issues influencing CS uptake in Ghana.

**Supplementary Information:**

The online version contains supplementary material available at 10.1186/s12978-026-02317-w.

## Background

Pregnancy and birth are crucial influences on the health of mothers worldwide. According to the World Health Organisation, many maternal mortalities are avoidable with the use of highly skilled obstetric care [1–4], including caesarean section (CS) [5], especially in resource-poor settings such as Ghana [6].

Caesarean section is a potentially life-saving surgical procedure that is essential for many women, such as those with obstructed labour and abnormal fetal presentation [7]. Like any other surgical procedure, CS also comes with potential complications, including death of the mother [8, 9]. It is associated with both short- and long-term risks, including maternal haemorrhage, infection, long recovery times, scar rupture and placenta accreta in future pregnancies [3, 9–11].

Several studies have reported that developed countries have been accruing increasing CS rates due to high societal acceptance of the procedure [9]. On the contrary, developing countries have only started to experience an increase in CS owing to minimal societal acceptance, even when there is a clear danger in pursuing vaginal birth [3, 9–12].

### The contextual evidence on CS in Ghana

In 2017, the Ghana Health Service (GHS) reported a CS rate of 16% [13]. Given that the WHO recommends a range of 10% to 15% per live birth, Ghana, as of 2017, had joined other countries that have exceeded this recommended range [7, 14]. In the Ghana Demographic Health Survey (GDHS) 2022 report, the CS rate had increased to 21% (for live births) [15], which matches the global CS rate [5, 7, 16].

As suggested by Miller et al. (2016), given the time frame, Ghana could have experienced the 'Too Much, Too Soon (TMTS)' phenomenon for CS [17] TMTS refers to the increasing trend of facility-based births that are associated with the over-medicalisation of childbirth, going beyond optimal use of CS. This overuse of medical interventions, even in uncomplicated pregnancies, may reduce or offset the potential health benefits gained from improvements in maternal and perinatal care. In Ghana, the rise in TMTS may be partly due to the over-medicalisation of pregnancies that do not require such intensive interventions [17, 18]. There are also major regional variations in CS rates across Ghana, as well as within health facilities [19, 20]. Research has found that geographical location, type of settlement or residence (urban or rural), demographic factors, socio-economic and socio-cultural factors are all linked to variations in CS rates across Ghana [6, 21–23]. This means there may still be pockets of under-use and other pockets of over-use, but what is less clear is why these rates vary widely between regions of Ghana. 

### Prevalence of CS reported by the 2014 and 2017 GDHS

**Fig. 1:**
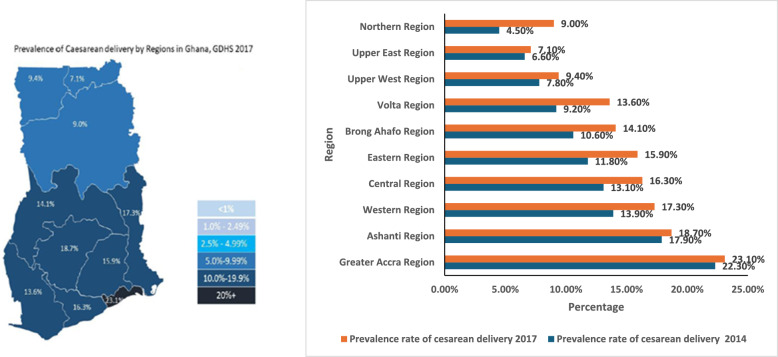
Map and comparative graph illustrating the prevalence of CS in Ghana in 2014 & 2017. Source: Data from GDHS, 2014 & 2017 [24, 25]

Figure 1 shows the comparative prevalence of CS rates by regions in Ghana in 2014 and 2017, respectively, which also shows significant changes in just 3 years. For instance, the Greater Accra region showed the highest rates of 22.3% and 23.1% and the Northern region had the lowest rate of 4.5% and 9.0% for 2014 and 2017, respectively [24, 25].

A scoping review is proposed to improve understanding of the variation in CS uptake across Ghana.

Scoping reviews are appropriate for mapping evidence on the current literature. They also allow for the discovery of evidence on relevant concepts, research gaps that inform policy and practice [26–28]. Hence, a scoping review was deemed the most appropriate and suitable method to explore, identify, chart, and discuss the available evidence on the prevalence of caesarean sections and factors influencing uptake across regions of Ghana.

## Conceptual framework

### The theoretical domains framework

The review adopted the Theoretical Domains Framework (TDF) as a framework to explore the barriers and facilitators affecting CS uptake in Ghana [29]. The TDF is used to categorise and explain why a certain behaviour occurs and to determine the factors influencing behaviour among health professionals and patients [29]. The TDF is made up of 14 theoretical domains and several constructs which represent a broad range of factors influencing behaviour. These domains include knowledge, environmental context and resources, skills, emotion, social influences, beliefs about capabilities, memory and attention decision processes, optimism, reinforcement, behaviour regulation, intentions, goals, beliefs about consequences, and social/professional roles and identity [30]. The TDF is appropriate for this review as it supports the exploration and identification of factors that influence the behaviour of healthcare professionals and women, particularly in relation to how these factors shape decisions around the uptake and delivery of CS [29]. Information on how the TDF domains were adapted for relevance to the current study is provided in the ‘Theoretical Domains Framework (TDF) Analysis’ section below in Table 8.

### Scoping review aims

The review aims to assess the prevalence of caesarean section across regions of Ghana and the barriers and facilitators to CS uptake in Ghana, to inform health improvement and future research on the optimum use of CS.

### Research question

The main research question was: What is known about the prevalence of CS and factors influencing uptake of CS in Ghana?

## Materials and methods

A scoping review was conducted. The Joanna Briggs Institute (JBI) methodological guidelines for scoping reviews [31] were followed, and the review was reported in line with the Preferred Reporting Items for Systematic and Meta-Analyses extension for scoping reviews (PRISMA-ScR) [31] (SI Appendix 1).

The protocol for this review was registered with Open Science Framework (OSF) on 04th April 2024 with registration DOI: 10.17605/OSF.IO/HCDER [32]. Subsequently, the protocol was published on 08th October 2025 [33]. There were no deviations from the registered protocol.

### Search strategy

Comprehensive literature searches were conducted in multiple electronic databases, including Ovid MEDLINE, PsycINFO, EMBASE, Web of Science and CINAHL(EBSCO), and the final searches were conducted on 4th and 5th April 2024. In accordance with the JBI guidance, the search strategy followed three steps [31]. An initial limited search (step one) was conducted with a librarian (MBi). The full Medline search strategy is reported in Table 1, and the search strategy employed for other databases is in SI Appendix 3. Additionally, journals and websites that publish information on CS were searched for published and grey literature. These include Google Scholar, Google, the WHO, World Bank, The Lancet, Ghana Health Service, UNICEF, AMDD, UNFPA, and the International Journal of Gynaecology and Obstetrics.

**Table 1:** Full Search strategy on Ovid Medline on 4th and 5th April 2024

Number	Search Terms	Results
1	''caesarean section*''.mp	21,487
2	''cesarean section*''.mp	68,255
3	''c-section*''.mp	2102
4	1 or 2 or 3	78,284
5	prevalence*.mp	913,617
6	rate*.mp	3,821,880
7	epidemiology*.mp	2,279,076
8	uptake*.mp	463,936
9	barrier*.mp	437,323
10	facilitator*.mp	41,054
11	factor*.mp	6,713,414
12	determinant*.mp	299,344
13	15 or 6 or 7 or 8 or 9 or 10 or 11 or 12	11,367,894
14	4 and 13	37,652
15	Ghana*.mp	17,854
16	West Africa*.mp	15,486
17	15 or 16	31,995
18	14 and 17	151
19	limit 18 to (English language and humans)	123

In Ovid Medline, the asterisks (*) are used as wildcards to represent zero or one character within aword or at the end of the word

## Results

**Fig. 2:**
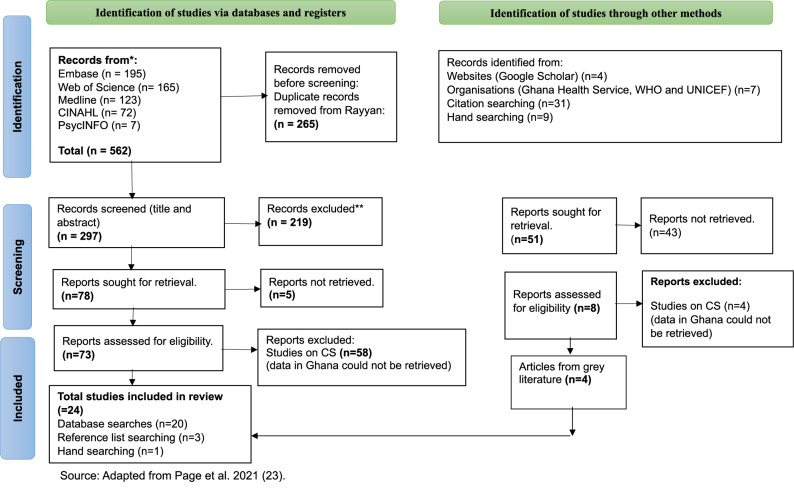
PRISMA flow diagram of database searches and other sources

A total of 562 papers were retrieved (see Fig. 2). After removing duplicate articles (265), a total of 297 papers were eligible for title and abstract screening. A total of 219 articles were excluded because they did not meet the inclusion criteria, and 78 articles were eligible for full-text screening. Five articles were further excluded at the full-text screening stage because the full texts were not available. During full-text screening, 58 articles were excluded because data specific to Ghana could not be identified. This left a total of 20 articles from databases that met the inclusion criteria. Out of 51 articles from other routes, such as website (Google Scholar), citation searching, 43 were excluded due to duplication with the database search. A total of 8 studies sourced from grey literature were full-text screened, and 4 of the articles reported eligible data on Ghana. Therefore, 24 eligible studies were included in this review.

Additionally, journals and websites that publish information on CS were searched for published and grey literature. These include Google Scholar, Google, the WHO, World Bank, The Lancet, Ghana Health Service, UNICEF, AMDD, UNFPA, and the International Journal of Gynaecology and Obstetrics. Handsearching was utilised to identify additional relevant papers. The searches were updated on 24 April 2024, and the findings were used to populate the PRISMA flow diagram (Fig. 2).

The study characteristics are presented using a table format displayed in Table 2, following the data extraction template in SI Appendix S2 (Tables 3, 4, 5, 6).

**Table 2:** Characteristics of the included studies

Evidence of the review’s findings							
**Author(s) & year**	**Setting**	**Aim**	**Study Design/** **Study Approach**	**Population & Sample Size**	**Data Sources/collection**	**Prevalence rates of CS in GH**	**Key Results**
*Apanga et al., (2018) [34]	Upper East-Ghana	To investigate obstetric, and socio demographic factors associated with CS in northern Ghana	Case–Control/Quantitative	Mothers (*n* = 450)	Routinely collected Secondary data	Not Reported	Pregnant women, with more than 4 antenatal visits, a gestational age between 37 to 40 weeks and a foetal weight of 4000 g or more had increased odds of undergoing a CS
*Manyeh et al., (2018) [21]	Greater Accra -Ghana	To examine the rate of CS delivery and to explore factors associated with the procedure in two rural districts in Ghana	Cross- sectional/Quantitative	Mothers (*n* = 4948)	*Secondary data from the DHDSS	6.6%	Study participants belonging to the richest wealth quintile were more than 2 times more likely to have CS. Maternal age (older women), educational level, parity, household socioeconomic status, district of residence, and level of education of household heads were all associated with CS delivery
*Seidu et al., (2020) [23]	**All Hospitals in Ghana	To determine the prevalence and assess the factors associated with caesarean delivery among childbearing women in Ghana	Cross- sectional/Quantitative	Mothers (*n* = 2742)	*Secondary data from GDHS	18.5%	Mothers’ advancement in age (45–49), parity (more than 4 deliveries), baby’s sex (male), sex of household head, type of settlement or residence (urban) geographical location or region (Southern or Northen Ghana) were factors found to influence CS in Southern Ghana
Rishworth et al., (2016) [6]	Upper West -Ghana	To explore women’s perceptions and experiences of CS in the Upper West Region of Ghana	Cross- sectional/Qualitative	Mothers (*n* = 170)	Focus group (*n* = 10) and in-depth interviews (*n* = 30)	Not Reported	Increase in rates of CS suggested improved access to skilled health care largely to promote maternal health outcomes. The authors advocate for improved health facilities in hard-to-reach communities with inadequate medical facilities to cater for the long-term risk of women undergoing CS and the long-term effects on their babies
Adu-Bonsaffoh et al., (2022) [22]	Greater Accra-Ghana	To explore mode of childbirth preferences and associated factors among pregnant women in Ghana	Cross- sectional/Quantitative	Mothers (*n* = 415)	Questionnaire data	14%	There was a clear preference for vaginal delivery (VD) (86%) by pregnant women. Majority (26%) attributed their preference for VD to it being the natural way of childbirth although a significant proportion preferred caesarean delivery (CD) (14%). The most common reason why women preferred to deliver by CS was a perceived medical indication such as that suggested by doctors’ remarks
*Okyere et al., (2022) [35]	**All Hospitals in Ghana	The analyse trends in the prevalence of birth by CS in Ghana from 1998 to 2014	Cross- sectional/Quantitative	Mothers (*n* = 15,432)	*Secondary data from GDHS	12.8%	The authors advocate for the reduction of the disparities found in having a CS birth, stating that women should undergo CS only when there is a genuine medical indication for it to reduce the risk with CS procedure in hospitals in Ghana

*UE* Upper East, *GA* Greater Accra, *UW* Upper West, *Hosp in Gh* Hospitals in Ghana, *CC* Case–Control, *CS* Cross- sectional, *QT* Quantitative, *QL* Qualitative, *Routinely collected retrospective Secondary data (Jan- May 2015), *DHDSS = Dodowa Health and Demographic Surveillance System *2011–2013, **All hospitals in Ghana = hospitals with doctors and operating theatres, *GDHS = Ghana Demographic and Health Survey (nationally representative data), 2014, *GDHS = Ghana Demographic and Health Surveys 1998–2014

**Table 3:** Characteristics of the included studies

Author(s) & year	Setting	Aim	Study Design/ Study Approach	Population & Sample Size	Data Sources/collection	Prevalence rates of CS in GH	Key Results
*Prah et al., (2017) [36]	Cape Coast -Ghana	To ascertain the prevalence and compare outcomes of elective and emergency CS among women who deliver at the University of Cape Coast Hospital, Ghana	Cross- sectional/Quantitative	*Mothers (*n* = 645)	Retrospective Secondary data	26.9%	The high CS rate reported was potentially attributed to the facility being a referral centre for other health facilities and thus receiving complicated pregnancies
*Owoo et al., (2017) [37]	**All Hospital in Ghana	To examine the factors correlated with CS delivery in Ghana, with an aim to make research-based policy suggestions	Cross- sectional/Quantitative	Mothers (*n* = 1284)	Routinely collected Secondary data	Not Reported	Maternity leave among women with high wealth status, advanced in age and lower maternal parity were important determinants of CS deliveries in Ghana. Child gender (male), more than 4 antenatal clinic visits and easy access to health facilities were significantly associated with CS. While antenatal care attendance at government institutions reduces the likelihood of CS, deliveries in government health facilities are more likely to be done by CS
*Dankwah et al., (2019) [38]	**All Hospitals in Ghana	To examine the use of CS in Ghana and the socioeconomic factors associated with it	Cross- sectional/Quantitative	Mothers (*n* = 4294)	*Secondary data from GDHS	11.4%	The study found that Ghana’s free maternal care policies, maternal age, parity, high educational level, and high wealth status of the mothers were significantly associated with CS delivery The percentage of CS delivery ranged from 5% of women in the poorest quintile to 27.5% of women in the richest quintile
*Gyaase et al., (2023) [39]	Bono East (Kintampo), Ghana	To determine the prevalence and factors influencing CS deliveries in the Kintampo Districts of Ghana	Cross- sectional/Quantitative	Mothers (*n* = 2,887)	*Secondary data from EN-INDEPTH	14.6%	CS was higher among women with high wealth status and women with a gestation between 37 to 40 weeks. A previous pregnancy loss and having more than 8 antenatal visits was associated with CS birth. Maternal age, educational level, marital status, parity, were other factors that were considered to increase a woman’s chances of undergoing a CS or VD

*C–C* Cape Coast

^**^All hospitals in Ghana = hospitals with doctors and operating theatres, BE (Kint) = Bono East (Kintampo), CS = Cross-sectional, QT = Quantitative, *Records of 645 women collected between January 2014 and December 2015, *GDHS Ghana Demographic and Health Survey (GDHS), 2014, EN-INDEPTH *Every Newborn–International Network for the Demographic Evaluation of Populations and their Health

**Table 4:** Characteristics of the included studies

Author(s) & year	Setting	Aim	Study Design/ Study Approach	Population & Sample Size	Data Sources/collection	Prevalence rates of CS in GH	Key Results
Asah-Opoku et al., (2023) [40]	Greater Accra-Ghana	To explore mothers’ knowledge, perceptions and factors influencing shared decision making about CS	Cross- sectional/Mixed method	Mothers (*n* = 177)	Questionnaires, Interviews using interview guide	Not Reported	Women had high level of knowledge regarding medical indications for their CS but had low level of awareness of shared decision making. Healthcare professionals attributed the willingness of mothers to be involved in shared decision making to their level of education. Health care professionals and post-partum mothers also attributed that the insufficient consultation time was a challenge to the shared decision-making process
*Banchani et al., (2020) [41]	**All Hospitals in Ghana	To investigate risk factors contributing to the decision to perform CS among women in Ghana	Cross- sectional/Quantitative	Mothers (*n* = 8645)	Secondary data from GMHS	13.4%	Most Ghanaian women (about 87%) preferred vaginal delivery to CS. Women (55%) who had undergone a recent CS had had an elective rather than an emergency section. Women with labour complications (prolonged/obstructed labour) were significantly more likely to have a CS. In addition, women with maternal complications, particularly prolonged/obstructed labour, were less likely to have an elective CS than those who had no such complications. Furthermore, wealthier women were significantly more likely to have an elective CS compared to their poorer counterparts
Konlan et al., (2019) [42]	Brong-Ahafo (Duayaw Nkwanta Hospital), Ghana	To identify the factors that influence women’s choice of elective CS in Duayaw Nkwanta Hospital	Cross-sectional/Quantitative	Mothers (*n* = 78)	Secondary data Questionnaire	42%	Post-CS women (37.2%) indicated CS is a pain-free method of birth, while 57.1% reported CS is safe for both mother and baby. Others (28.2%) chose CS based on a friend’s advice, and 19.2% on religious advice. There was a weak positive correlation between age of respondents and the number of CS women undergo. The average monthly income of respondents and the number of times of having a CS birth had a significant positive correlation
*Mireku-Gyimah (2015) [43]	Greater Accra-Ghana	To identify the sociodemographic and obstetric predictors of CS in a major referral health facility in Accra, Ghana	Case control/Quantitative	Mothers (*n* = 5,640)	Routinely collected Secondary data from health facility records	Not Reported	Having a CS was associated with maternal age (35–49 years), higher level of education, obstetric history and higher infant weight (< 3.5 kg). There was no significant association observed between mode of delivery and gestational age or parity. Lower odds were found among women with secondary education and antenatal care non-attendants attributed to lack of knowledge and misconceptions of CS uptake among women. There was an association between health care providers targeting wealthier clients to have a CS for profit gains
*Zethof et al., (2023) [44]	**All Hospitals in Ghana	To compare CS rates between women with live births and stillbirths and identify socioeconomic and pregnancy-related factors associated with CS in stillbirths	Population-based Cross-sectional/Quantitative	Mothers (*n* = 17,138)	Routinely collected Secondary data Interviews using questionnaires	9.9%	The CS rate in women with stillbirths or very early neonatal deaths were 19.3% compared with 9.6% in women with live births who survived the first day. There was an association between higher household wealth and educational levels with increased risk of CS in both study groups, with no statistically significant difference in effect

*GA* Greater Accra, ** All hospitals in Ghana = hospitals with doctors and operating theatres, B-A(DN) Brong-Ahafo (Duayaw Nkwanta Hospital), CS = Cross-sectional, CC = Case control QT = Quantitative, MM = Mixed method, * GMHS = Ghana Maternal Health Survey (nationally representative data), 2017 conducted by the Ghana Statistical Service (GSS), Ghana Health Service (GHS) and Macro ICF, *Routine data from the research unit of a major referral health facility in Accra, Ghana between January and October 2017

**Table 5:** Characteristics of the included studies

**Author(s) & year**	**Setting**	**Aim**	**Study Design/** **Study Approach**	**Population & Sample Size**	**Data Sources/collection**	**Prevalence rates of CS in GH**	**Key Results**
*Bam et al., (2021) [45]	District Hospital in Ghana	To determine the factors that influence women's decision-making and the duration of the decision-making process to accept primary or repeat elective CS in a district hospital in Ghana	Cross- sectional/Quantitative	Mothers (*n* = 163)	Questionnaire data	33.7%	Women's decision to accept a CS was influenced by husband/partner and relatives (39.3%). This highlights the need to involve relatives during the antenatal care period to help younger women make timely decisions and preventable complications. A woman accepting to undergo a CS was also because of baby's life at risk (24.5%), previous CS, knowledge about CS (19.6%). A woman’s age (older women) and parity were other influencing factors
*Adu-Bonsaffoh., (2022) [46]	Greater Accra (Ghana & Dominican Republic)	To explore the characteristics of women receiving emergency CS using a new, validated definition in Ghana and the Dominican Republic	Cross- sectional/Quantitative	Mothers (*n* = 653)	Routinely collected Secondary data	14.0%	A woman’s advanced age (35–49 years), gestation above 37 weeks on referral was associated with having a CS. Emergency CS was higher among mothers without a previous CS and mothers having their first child, and lower among mothers with prior birth. CS was lower among mothers with preterm babies in both Ghana and the Dominican Republic
*Islam et al., (2022) [47]	Sub-Saharan Africa including hospitals in Ghana	To examine the prevalence and determinants associated with caesarean delivery in Sub-Saharan Africa countries	Cross- sectional/Quantitative	Mothers (*n* = 3091)	Secondary data from DHS data from 2008–2016	15.6%	CS in public health facilities was 15.6% in Ghana. The odds of having CS increased with maternal age between 35–49 years. Women with a significant number of antenatal visits had reduced odds of getting a CS birth. Women from richer households and expecting a male child, with predicted weight more than 3.5 kg had increase odds of CS birth if they attended a public health facility. While women attending a private health facility with multiple births and who had decision making power had increased odds of CS birth
*Boatin., (2017) [48]	Greater Accra—Ghana	To describe facility-based decision-making for women with one prior CS delivery in a resource-limited setting	Cohort study/Quantitative	Mothers (*n* = 1247)	Secondary data	12.0%	Of the 1247 women who had one prior CS, 377 (30.2%) underwent non-emergent repeat CS delivery (RCD), 439 (35.2%) had emergency repeat CS delivery (EMCD) and 431 (34.6%) had vaginal birth after trial of labour after CS (TOLAC). The study reported that the prevalence rate of CS likely reflects that most of the women who presented to deliver at the facility (urban tertiary care centre) were directed towards CS while less than a third of the women were supported to have a supervised trial of labor

District hospital in Ghana, *GA* Greater Accra, *SSA* Sub-Saharan Africa, *Hosp in Gh* Hospitals in Ghana, *CS* Cross- sectional, *QT* Quantitative, *A descriptive cross-sectional study, * Routinely collected Secondary data from surgical register at the labor ward of the Korle-Bu Teaching Hospital (KBTH) in Accra, Ghana between June – August 2021,, *Secondary data drawn from the clinical database of the hospital (One year retrospective study of women with one prior CD delivering at Korle-Bu Teaching Hospital (KBTH), DHS = Demographic and Health Survey (DHS) data from 36 Sub-Saharan Africa, * DHS = Demographic and Health Survey from 34 Sub-Saharan Africa between 2008–2016

**Table 6:** Characteristics of the included studies

Author(s) & year	Setting	Aim	Study Design/ Study Approach	Population & Sample Size	Data Sources/collection	Prevalence rates of CS in GH	Key Results
*Gandau et al., (2019) [49]	Upper West, Ghana	To determine maternal perceptions about CS deliveries and their role in reducing neonatal mortality at a regional and a district hospital in the Upper West Region of Ghana	Cross sectional, Quantitative	Mothers (*n* = 416)	Questionnaires data	26%	A total of n = 348 (87.4%) women preferred spontaneous vaginal delivery though n = 281 (73%) of the mothers agreed to undergo a CS if it were necessary. Mothers not wanting a CS was n = 148 (51.8%) due to the long recovery time. Inadequate knowledge about CS led *n* = 180 (45.1%) of the mothers to conclude that CS cannot promote child survival. Women, *n* = 85 (21.6%) believed CS had adverse effects on child survival. The factors that contributed to low knowledge of CS was largely because mothers had no formal education, no occupation and were aged less than 19 years
*Kondor (2023) [50]	Greater Accra—Ghana	To determine the prevalence of CS at LEKMA Hospital in Ghana and the demographic and clinical history of mothers as predictors for the prevalence	Cross sectional, Quantitative	Mothers (*n* = 598)	Routinely collected Secondary data	40.6%	Sociodemographic and the clinical history of a pregnant woman linked to the mode of delivery. Increase in gestational age and parity reduced the risk of CS. Lower level of education and mothers’ age above 36 years increased a risk of CS delivery
*Darteh (2020) [51]	**All Hospitals in Ghana	To examine the characteristics of women in their reproductive age delivering via elective CS in Ghana	Cross sectional, Quantitative	Mothers (*n* = 11,309)	Secondary data from (GMHS)	12.4%	Maternal age (40–44 years), richer status, higher level of education, place of residence (urban) religion, the rate of recurrence in reading, listening to radio and watching Television were significantly associated with CS birth. There were high proportions of women seeking CS without any maternal and foetal indications compared to women with indications
*Alhassan (2022) [7]	Tamale—Ghana	To examine the prevalence of CS and the socioeconomic factors that predict CS delivery	Cross sectional, Quantitative	Mothers (*n* = 2673)	Secondary data from MICS	15.7%	The socioeconomic predictors of CS in urban Tamale were age, place of residence, income status and, health insurance coverage. Babies with predicted normal birth weight were less likely (0.6 times) to be delivered through CS compared with those with low birth weight. Mothers in the richest index quintile were more likely (2.4 times) to experience CS delivery compared with those in the poorest index quintile. Mothers 25–34 years were more likely (1.6 times) to have birth by CS compared with those 15–24 years. In addition, women 35 years and above were more likely (2.1 times) to experience CS delivery when compared with those age 15–24 years. However, rural women in Tamale were less likely (0.7 times) to experience CS delivery compared to their urban counterparts

*UW* Upper West, *GA* Greater Accra, *CS* Cross- sectional, *Tle* Tamale

^**^All hospitals in Ghana = hospitals with doctors and operating theatres QT = Quantitative, CohtS = Cohort study,

^*^Facility-based retrospective data of 2019, GMHS = Ghana Maternal Health Survey (2017), * MICS = Ghana multiple indicator cluster survey between October 2017 and January 2018

### Eligibility criteria

The criteria used to assess the eligibility of papers for the scoping review is summarised in Table 7 (below).

**Table 7:** Inclusion and exclusion criteria

Inclusion criteria: Studies that:
◦ Include findings on women who have given birth in Ghana, through CS (either elective or emergency CS) or vaginal birth
◦ Conducted in any setting, including home or hospital
◦ Primary qualitative, quantitative, mixed methods studies, as well as systematic and scoping reviews, and grey literature
◦ Published in English
◦ Reported on the prevalence of CS, and/or the barriers, and facilitators influencing the uptake of CS with data specific to Ghana
Exclusion criteria
◦ Any studies or reports that contained data on other countries, as well as Ghana, where data specific to Ghana could not be extracted separately

### Screening of studies selected

Title and abstract screening were conducted independently by two reviewers (SM and JD). The third and fourth reviewers (LL and MB) acted as arbitrators where agreement had not been reached by the first and second reviewers.

### Data extraction

The Rayyan web tool was used to conduct the data extraction following a data extraction template adapted from the JBI Evidence Synthesis manual [52]. The data extraction template was tested by the reviewers during the protocol stage to independently pilot the template on three randomly selected papers. The primary researcher (SM) conducted the data extraction.

### Data analysis

#### Theoretical Domains Framework (TDF) analysis

Two behaviours were the primary focus of the TDF analysis: (1) healthcare professionals’ recommendations for CS, and (2) women’s decisions to undergo either a CS or a vaginal delivery.

A codebook based on the TDF was developed (See SI Appendix 4). Eligible study findings were coded according to the relevant TDF domains, identifying domain-specific barriers and facilitators influencing the uptake of CS in Ghana. The coding took into consideration the domain, construct, decision rules and direct quotes to build up the codebook. This was reviewed and updated iteratively with the supervisory team to ensure robust and relevant coding. The data was presented in a Table format.

A sample of the coding rules adopted and used in this scoping review is summarised in Table 8 (below).

**Table 8:** Sample of the TDF Codebook describing selected domains

TDF Domains:
Environmental context and resources: content was coded under this domain when the environment impacted on the decision of a HCP to recommend a CS (e.g. due to available skillset) and if the availability of resources affected a mother’s decision for a CS
Social Influences: content was coded under this domain when social or interpersonal factors, such as religious beliefs, influenced either a woman’s decision to undergo a CS or HCP’s decision to recommend one
Beliefs about consequences: this domain was coded where there were perceived benefits or risks of a CS which influenced HCP or women’s decisions regarding CS
Emotion: content was coded to this domain when mothers expressed anxiety, fear or relief in relation to the concept of undergoing a CS. When HCPs described emotional responses, such as pressure, frustration, or concern, arising from cultural and societal expectations, this content was also coded under the Emotion domain
Knowledge: content was coded to this domain when it related to mothers’ knowledge of undergoing CS, including factors such as pain management, recovery time and its side effects. For HCPs, content was coded to this domain in relation to the knowledge HCPs have regarding the indications for recommending a CS
Memory and attention decision processes: the content was coded to this domain when mothers’ reflections of their experiences on previous childbirth reflect on their current mode of birth and/or are impacted by the experiences of family and friends

#### Data extraction, coding and presentation process

Data extraction and coding were led by SM. Additionally, LL carried out double coding of a sample of the data and compared this against the TDF codebook. The team held sequential meetings to discuss the applicability of the data to the TDF and verify the accuracy of the coding.

**Fig. 3:**
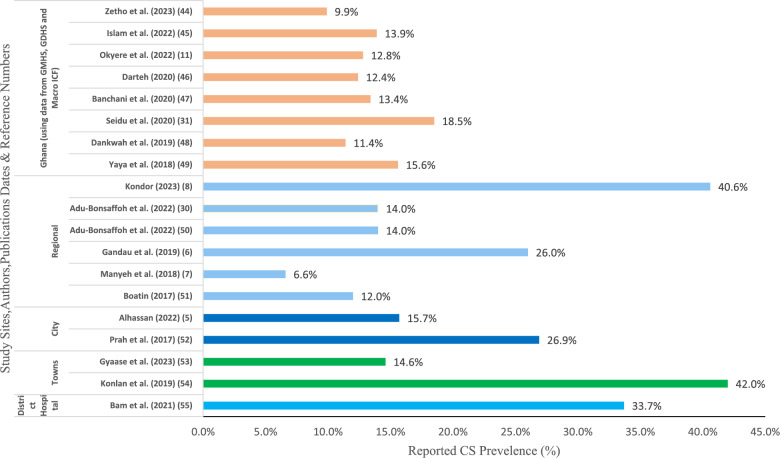
Bar chart showing papers which reported on caesarean rates in Ghana. *Notes:* GMHS = Ghana Maternal Health Survey, GSS = Ghana Statistical Service, GHS = Ghana Health Service.

A map and comparative graph (see Fig. 3) using data from the 2014 and 2017 Ghana Demography and Health Survey (GDHS) were utilised to illustrate the recent prevalence rates of CS in and across Ghana. A comprehensive descriptive summary of the data extracted was reported [53].

#### Data extraction of prevalence rates

Data on the prevalence of CS rates were extracted by mapping them within a table according to the year of the study and across each region in Ghana. The results were presented graphically in a bar chart format to understand the trend of the prevalence of CS rates across regions of Ghana (Fig. 3).

## Results

### Studies reporting on CS prevalence

Nineteen studies reported the prevalence rates of CS [21–23, 35, 36, 38, 39, 41, 42, 44–51, 54, 55] (Fig. 2). The highest prevalence rate recorded was 40.6% in the Greater Accra region (Southern Ghana) [50]. This was a facility-based prevalence rate. The lowest recorded prevalence rate was 6.6% [21] across two rural districts of the Greater Accra region of Ghana.

All the included studies reported on their sample size. These ranged from 78 to 15,432 women participants. Table 2 provides a summary of the review findings of the included studies as well as the key results and conclusions reached by the primary authors.

### Descriptive data summary

Quantitative data from eligible studies (Fig. 2) were extracted and graphically presented to demonstrate the reported prevalence of CS uptake across Ghana and how it varies by region.

Figure 2 shows the reviews’ studies that reported on Prevalence of CS in Ghana.

The horizontal bar chart in Fig. 2 presents the descriptive CS prevalence that was reported in the included studies.

### Summary of the study characteristics

Table 2 provides a summary of the characteristics and key findings of the studies included in this review. The publication year of the studies ranged from 2015 to 2023. The studies were all conducted in urban and/or district (rural) settings in Ghana. The geographical settings of the included studies are as follows: Ghana (*n* = 9), [14, 23, 35, 38, 41, 44, 47, 51, 55] Greater Accra (*n* = 7), [8, 10, 30, 33, 38, 44, 51], Upper West (*n* = 2), [6, 54] Upper East (*n* = 1), [34], Cape Coast (*n* = 1) [36], Tamale (*n* = 1), [49], Kintampo (*n* = 1), [39], Duayaw Nkwanta (*n* = 1), [42] and district hospital (*n* = 1), [45]. There were twenty-one cross-sectional studies [14, 21–23, 34–36, 38–42, 44–47, 49–51, 54, 55] two case–control studies [34, 43], and one cohort study [48]. Of the 24 studies included, 22 were quantitative studies [14, 21–23, 34–36, 38, 39, 41–51, 54, 55], with one qualitative study [6] and one mixed-method study [40].

### Conclusions drawn on prevalence rates

The conclusion drawn on the prevalence of CS in most studies was that higher rates in urban tertiary health facilities reflect these being referral centres for other health facilities and thus care for complicated pregnancies with a high chance of CS [21–23, 35, 36, 38, 39, 41, 42, 44–51, 54, 55].

Other studies concluded that Ghana’s Free Maternal Healthcare Policy (FMHCP) was one major contributor to increased CS prevalence [56, 57]. The aim of the FMHCP was to remove the financial burden of accessing maternal and neonatal health services, including CS births, for all pregnant women across Ghana for three months. Subsequently, mothers are required to enrol their children in the scheme [56, 57] to continue to receive these health care services.

## Findings from the TDF analysis

### Summary of eligible studies

Ten eligible studies were TDF-coded [6, 22, 34, 40–42, 45, 47, 51, 54]. The most studies that contained most details on the barriers and facilitators across all the coded domains were Rishworth et al. (2016) [6], a qualitative study and Asah-Opoku Kwaku [40], a mixed-method study, appearing seven and six times, respectively. Seven of the 14 TDF domains (*environmental context and resource, social influence, belief about consequence, emotions, knowledge, belief about capabilities and Memory, attention, and decision processes)* were identified as relevant.

The remaining seven domains *(skills*, *optimism, reinforcement, behaviour regulation, intentions, goals and social/professional role and identity)* were not coded; information presented under these domains was not explicit within the papers in terms of how they impacted the decision to perform or undergo a CS. See Table 8 for an excerpt of the TDF codebook and examples of how the domains were applied in the analysis. The full TDF codebook is available to view in S1 Appendix 4.

### Barriers and facilitators to the uptake of CS

The most frequently reported barriers to the uptake of CS in Ghana were coded under the TDF domains: ‘Environmental Context and Resources’, ‘ Social influences’, ‘Beliefs About Consequences’, ‘Emotions’, and ‘ Knowledge’. The most frequently occurring facilitators that were reported to encourage the uptake/recommendation of CS were categorised under the ‘Beliefs About Capabilities’, ‘Memory, Attention and Decision Processes’ domains.

The domains presented below are arranged in ascending order according to the number of times they were coded across the 10 eligible studies.

### Environmental context and resources

The Environmental Context and Resources (ECR) domain was the most frequently coded domain across seven studies [6, 34, 40, 41, 45, 47, 54].

ECR was identified as a barrier as well as a facilitator, capturing logistical factors that influenced decisions to undergo or perform a CS. These included the availability of healthcare facilities, time constraints in emergency settings, and financial factors.

For example, limited time to discuss delivery options in emergencies influenced whether a CS was performed or not as this was identified as a barrier to making informed decisions about undergoing a CS.

“*Because they [healthcare professionals] were in a hurry I didn’t talk [ask]. If I talk (asked), they might tell me oh…’’* Mother participant during interviews using a questionnaire, Asah-Opoku et al. (2023), [40].

Institutional policies and guidelines influencing the prevalence of CS in Ghana also appear to act as a facilitator for recommending CS to women [40]. These policies and guidelines give a clear indication of women who meet the criteria for a CS [34, 54]. Such women stand a higher chance of having another CS in a subsequent pregnancy. Therefore, adhering to these policies and guidelines acts as a facilitator to CS uptake [34, 54].

Taken together, the studies coded under this domain highlight how structural and contextual factors in the healthcare system can impact optimal use of CS.

### Social influences

The domain looked at how family members' opinions or experiences impacted mothers' decision-making regarding CS. Likewise, healthcare professionals' recommendations or preferences that affect mothers' choices were also explored in relation to this domain.

This domain was the second most frequently coded domain [6, 40, 42, 51, 54]. Women’s decision on the mode of birth was influenced by experiences shared by family, friends and HCPs' birthing preference:

*“Women still are scared when they hear the word operation. So scared that some go and see pastors and the pastors would convince them they would deliver on their own (and these women wait) until complications set in.”* Senior Resident (healthcare professional) during interviews using a questionnaire in Asah-Opoku et al. (2023).

The domain also found that societal norms, cultural beliefs and religious beliefs also shape attitudes towards CS.

In sum, the coded studies demonstrate that women’s decisions to undergo a CS can be influenced by barriers such as cultural norms and beliefs.

### Beliefs about consequences

This domain was used to understand what the perceived benefits, risks and consequences were for choosing a CS according to mothers and healthcare professionals, and how these impact decision making. This included weighing the short-term benefits, such as convenience, against the long-term risks, such as complications in subsequent pregnancies. Four studies were coded under this domain [6, 40, 45, 54]. An example of the weighing up of risk is presented in the quote below:

… *I think the C-section is given to save your life and the child’s life. Even if the child dies, to save the mother, so it’s a lifesaving technique’’*. Mother participant during Focus Group discussion in Rishworth et al. (2016).

Mothers saw the long-term risks of CS as a barrier, but would accept CS if they believed it to be lifesaving in a complicated pregnancy.

### Emotion

The review employed this domain to identify the emotions mothers experience when considering a CS (e.g., anxiety, fear, relief). Also, the review sought to understand how healthcare professionals' emotions concerning culture, societal norms, litigation and profit gains impact their views on CS.

Four studies could be coded under the ‘ Emotion’ domain, with relevant data [6, 22, 40, 42]. In the study of Asah-Opoku et al. (2023), women expressed their fear of having a CS procedure rather than having a vaginal birth, and some women expressed that they did not want to undergo the CS procedure for fear of losing their lives.

The barriers coded under this domain show how women base their decision not to undergo a CS on fear based on both their previous experiences and those gathered from family and friends.

### Knowledge

This domain highlighted how women’s knowledge about CS and the implications of having a CS (e.g., side effects, recovery time, pain management) influences the mode of birth. Furthermore, it highlighted the role of knowledge about indications for CS among mothers and healthcare professionals. The review coded three studies [6, 40, 54] under the ‘knowledge’ domain.

Some women were not satisfied with the amount of information they received before undergoing the CS procedure, suggesting that a lack of knowledge acted as a barrier to informed choice:

“... *actually, I’d have loved to know like, the complications it comes with. The bad, the negative sides of it. I’d have loved to know before….”* Mother participant during focus group discussion in Asah-Opoku et al., (2023)

### Beliefs about capabilities

Content was coded to this domain if HCPs demonstrated confidence in themselves or other HCPs to perform CS or manage labour and potential complications. Content was also coded to this domain in relation to the confidence mothers showed to decide on their own to have a CS. The content was further coded where demonstrating skills, knowledge and confidence were necessary for HCPs and mothers to take control of their decision-making regarding CS uptake.

Three studies [6, 40, 45] were identified and coded under this domain.

The perceived skills and capabilities of HCPs to perform and manage childbirth according to mothers were coded as a facilitator to CS uptake:

*‘’The C-section is much more difficult to do than normal delivery. There are complications that develop later in labour, so if you don’t take care of it, the person may die. So, if they’re performing a C-section, the doctors are aware and think it’s right for the woman’’.* Mother participant during in-depth interview. Rishworth et al. (2016).

Put together, women would accept to undergo a CS if they had the confidence and were empowered to make the decision on CS.

### Memory, attention, and decision processes

The goal of the coding rule for this domain was to assess whether HCPs and mothers based their decisions on past experiences. For example, whether HCPs recommending a CS acted based on previous experiences of performing the procedure and whether women made decisions about childbirth mode based on their own past experiences or those of family and friends. Content was coded to this domain in two studies [6, 45].

To illustrate, women considered their own past experiences of childbirth and those of family and friends to inform their decision to undergo a CS. Furthermore, the rule was applied to understand from both the perspective of women and HCPs the positive or negative experiences they have of either vaginal birth or CS and how that influenced their preference, and the perceived risk and benefits associated with each option.

“…*there were women who accepted the CS because they felt they did not have an alternative to the CS” because they had previous CS and were no longer afraid to undergo CS and those who consented because they felt their life was at risk”*. Senior Resident (healthcare professional), Bam et al., (2021).

In conclusion, the data coded in this domain indicate that previous childbirth influenced the uptake of CS.

A comprehensive table with example content coded under each TDF domain is available in SI Appendix:5.

## Discussion

### Summary of main findings

While the evidence on the prevalence of CS and factors influencing uptake in Ghana, some studies provided data which could be coded under TDF domains, but overall, there was relatively little data of relevance. Although all ten papers were coded using the TDF, only one qualitative study [6], provided sufficiently rich data to enable comprehensive mapping against the framework. This highlights a gap in the current literature and underscores the need for more in-depth qualitative research on the behavioural factors influencing CS uptake. Therefore, the impact of social influencers, knowledge, emotions, Beliefs About capabilities. Belief About Consequences and Optimism are domains that could be of interest to explore further.

This scoping review also explored the prevalence of CS and the factors influencing uptake in Ghana. Substantial variation in CS prevalence was identified across Ghana, with rates ranging 26% and 46% between the North and the South, respectively [21, 49, 50, 54].

In another West African country with similar health services/social challenges, Emmanuel et al. (2014) reported CS data from the 2013 Nigeria Demographic and Health Survey (NDHS), a nationally representative cross-sectional survey by the National Population Commission of Nigeria. This found a CS rate of 2.1% [58], significantly lower than institutional based regional rates reported in earlier studies: 11.3% in the North-West (2013); 18.8% in the South-East (2014); and 40.1% in the South-West regions (2016) [59–61]. The 2013 NDHS also revealed variations in CS rates within Nigeria: 4.7% in South-East; 0.6% in the North-West; and North-East [58]. A major contribution to regional variation is reported to be that 46.5% of women across Nigeria do not use antenatal care, including 61.1% in rural Nigeria and 22.4% in urban areas [62]. Lack of access to maternal health services were also observed in the study by Perpetus Ibekwe (2011), which reported that poor access to health facilities and insufficient health care professionals resulted in low coverage of maternal services [63]. Similarly, studies in Ghana suggest that increasing variation in CS rates from South to North could be attributed to the lack of health facilities and healthcare professionals’ availability in the Northern region [6, 22, 23]. In the study of Chigbu et al. (2007) and Adewuyi et al. (2018), factors linked to variations in the uptake of CS in regions in Nigeria included the lack of knowledge and counselling to support pregnant women to make informed decisions about their mode of birth, especially when necessary to save lives [62, 64]. These findings align with this review, which indicated that some women are not well informed about the implications of CS, suggesting that having adequate knowledge would have been an important factor influencing their decision to undergo a CS [6, 40, 54].

Quantitative factors positively associated with CS uptake in Ghana were: Maternal age (older women), high parity, baby sex (male), male household head, urban residence, geographical location (south), educational status of women/household head (secondary education), perceived medical indication, health insurance coverage (free maternal healthcare policy), labour complications (prolonged/obstructed labour), maternal complications and high household socio-economic status [34, 35, 56, 65, 66].

### Key findings from the TDF

Barriers to the uptake of CS in Ghana were coded under the following domains: ‘Environmental Context and Resources’, ‘ Social influences’, ‘Beliefs About Consequences’, ‘Emotions’, and ‘ Knowledge’. The facilitators identified included ‘Beliefs About Capabilities’, ‘Memory, Attention and Decision Processes’ domains. While all 14 TDF domains were considered during coding, the limited richness of data in some studies meant that seven TDF domains had no data coded to them.

### Key factors influencing CS uptake in the context of the wider literature

#### The barriers

Under the environmental context and resources domain, the dominant barriers included time constraints in emergency settings and the availability of health facilities. Time constraints in particular hindered efforts of both healthcare professionals and patients to fully engage in discussions during emergencies. This was identified in seven of the eligible studies [6, 34, 40, 41, 45, 47, 54]. Two of the included studies acknowledged that the failure to follow polices and guidelines acts as a barrier to CS uptake [34, 54]. The study by Francke et al. (2008) [67] advocate that it is not enough for policymakers to disseminate guidelines and expect the implementers to adhere, but that this should come along with adequate resources, along with clear and effective directives for their active implementation [67].

A further barrier in this review and previous systematic reviews was social influences [6, 40, 42, 51, 54]. Anxiety from the patient's families was most apparent in other studies [68–70], but in this review, healthcare professionals expressed their challenge in complying with guidelines recommending CS due to pregnant women’s family opinions [40].

#### The facilitators

The facilitators identified in this review indicated that the uptake of CS is influenced by both healthcare professionals and women [71] According to Smith et al. (2023), Beliefs About Capabilities’ and ‘Memory, Attention and Decision Processes’ domains empower patients to ask the right questions and make decisions for themselves based on their knowledge of one or both birth modes [72].

### The review findings in the context of the wider literature

Quantitative and qualitative data highlighted that social factors are critical influences on the uptake of CS in Ghana. This is consistent with findings of Bam et al. (2021), who reported that social factors influencing the uptake of CS in a district hospital in Ghana included the role of relatives, pregnant women not having an alternative to CS, and women’s previous CS experience [45]. Pregnant women depended on social support and considered the experiences and the opinions of family and friends in deciding their mode of birth [45] It has been recognised that most women in developing countries, including Ghana, do not have complete autonomy over their health due to cultural norms/beliefs, family/relatives, and place of residence [58, 73–75]. According to Daniel et al. (2016) in their cross-sectional study conducted in Northwestern Nigeria, it is the prerogative of husbands/partners, mothers-in-law, and family heads to make decisions concerning women’s mode of birth [61].

### Strengths, weaknesses and future directions

The major strength of this scoping review is the use of a conceptual framework (TDF) to guide the review. This ensured a comprehensive identification of potential barriers/facilitators affecting CS uptake in Ghana [29] from the perspective of healthcare professionals’ recommendations of CS to women and women’s choices regarding undergoing a CS or vaginal birth. This approach is novel in this area of research specific to Ghana. Adhering to the Joanna Briggs Institute methodological guidelines [53] for scoping reviews ensured a robust methodological approach. Considering both the ‘what?’ and the ‘why?’ together, the review is strengthened through our combination of research questions to address the CS prevalence and underlying factors affecting the uptake of CS.

However, our findings are not without limitations. The review did not consider an assessment of the methodological quality of the included studies, as this was not within the remit of a scoping review. Due to time and resource constraints, authors were not contacted for full-text papers where only the abstracts appeared relevant to this review’s objectives. In addition, relevant papers from pre-print repositories were not searched. Regarding the data extraction for this review, our methodological quality of the studies included in this review had limitations. Some papers were not clear on providing key study information, such as the region [14, 23, 38, 41, 44, 45, 47, 51, 55, 76]. It was not possible to apply the TDF to studies that did not contain enough information to judge whether the TDF domains were relevant or not (according to the coding rules we developed for this review).

A lack of qualitative studies on factors that influence the uptake of CS needs attention. Qualitative studies aid understanding of the views and perceptions held by women, their social networks and healthcare providers regarding the uptake of CS. This underpins the need for more qualitative work in this area. Moreover, some of the information presented in the papers reviewed were not an ‘in-depth’ account of women’s decision-making process or that of HCPs regarding whether to have a CS or not. Finally, due to time constraints, the review did not include data from publicly available datasets on Ghana’s CS rates, which could have provided a more in-depth understanding of the prevalence rates of CS in the country.

### Implications for future research

The findings of this scoping review give justification for future research into understanding the barriers and facilitators to the uptake of CS in Ghana to inform health improvement and equity, including in-depth qualitative exploration of the factors involved.

Furthermore, the majority of the quantitative papers included in this review suggest that a systematic review with meta-analysis could be conducted to synthesise the evidence on associations between clinical and demographic characteristics and CS uptake to increase precision in the results. Conducting a meta-analysis was beyond the scope of this scoping review. Furthermore, we recommend future data synthesis (mixed methods triangulation) to increase understanding of the qualitative and quantitative data on CS uptake in Ghana.

## Conclusion

This scoping review has given an overview of the published literature, which enhances the understanding of prevalence and the barriers and facilitators to CS uptake in Ghana. In this review, we identified a huge variation in CS rates across Ghana, and that the following domains act as barriers to the uptake of CS: *Environmental Context and Resources; Social Influences; Beliefs about Consequences; Emotions and Knowledge.* Facilitators of the uptake of CS included *Beliefs about Capabilities and Memory, Attention, and Decision Processes*. Despite enormous variation in CS uptake across Ghana and several reports on clinical and demographic factors associated with CS uptake, there is very little in-depth evidence published on the nature of the barriers and facilitators to CS uptake; thus, future qualitative research is warranted.

## Supplementary Information

Additional file 1: SI Appendix 1: Preferred Reporting Items for Systematic Reviews and Meta-Analyses Extension for Scoping ReviewChecklist. SI Appendix 2: Data Extraction Template. SI Appendix 3: Searches planned for each database and rationale. SI Appendix 4: Summary of the code book adopted. SI Appendix 5: Evidence of the TDF-specific domains coded by studies

## Data Availability

No datasets were generated or analysed during the current study.

## Abbreviations

CS: Caesarean Section
The WHO: World Health Organisation
PRISMA-ScR: Preferred Reporting Items for Systematic and Meta-Analyses extension for scoping reviews
TDF: Theoretical Domains Framework
GHS: Ghana Health Service
GDHS: Ghana Demographic Health Survey
TMTS: Too much, too soon
JBI: The Joanna Briggs Institute
SI: Supplementary Information
FMHCP: Free Maternal Healthcare Policy
ANC: Antenatal Clinic
NDHS: Nigeria Demographic and Health Survey
ECR: Environmental Context and Resources
HCP(s): Healthcare Professional(s)
