# Supplementary material for: Evidence on prevalence of caesarean sections and factors influencing uptake in Ghana: a scoping review
Source: Reprod Health. 2026 Apr 14;23:109. doi: 10.1186/s12978-026-02317-w (PMC13220613; doi:10.1186/s12978-026-02317-w)
Supplement: Supplementary file 1 — Additional file 1: SI Appendix 1: Preferred Reporting Items for Systematic Reviews and Meta-Analyses Extension for Scoping ReviewChecklist. SI Appendix 2: Data Extraction Template. SI Appendix 3: Searches planned for each database and rationale. SI Appendix 4: Summary of the code book adopted. SI Appendix 5: Evidence of the TDF-specific domains coded by studies [file 12978_2026_2317_MOESM1_ESM.docx]

**Supporting Information**

SI Appendix 1: Preferred Reporting Items for Systematic Reviews and Meta-Analyses Extension for Scoping Review (PRISMA-ScR) Checklist.

SI Appendix 2: Data Extraction Template

SI Appendix 3: Searches planned for each database and rationale

SI Appendix 4: Summary of the code book adopted

SI Appendix 5: Evidence of the TDF-specific domains coded by studies

Appendices

SI Appendix 1: Preferred Reporting Items for Systematic Reviews and Meta-Analyses Extension for Scoping Reviews (PRISMA-ScR) Checklist

| **SECTION** | **ITEM** | **PRISMA-ScR CHECKLIST ITEM** | **REPORTED ON PAGE #** |
| --- | --- | --- | --- |
| **TITLE** | | | |
| Title | 1 | Identify the report as a scoping review. | Page 1 |
| **ABSTRACT** | | | |
| Structured summary | 2 | Provide a structured summary that includes (as applicable): background, objectives, eligibility criteria, sources of evidence, charting methods, results, and conclusions that relate to the review questions and objectives. | Page 3 |
| **INTRODUCTION** | | | |
| Rationale | 3 | Describe the rationale for the review in the context of what is already known. Explain why the review questions/objectives lend themselves to a scoping review approach. | Page 4-5 |
| Objectives | 4 | Provide an explicit statement of the questions and objectives being addressed with reference to their key elements (e.g., population or participants, concepts, and context) or other relevant key elements used to conceptualize the review questions and/or objectives. | Page 6 |
| **METHODS** | | | |
| Protocol and registration | 5 | Indicate whether a review protocol exists; state if and where it can be accessed (e.g., a Web address); and if available, provide registration information, including the registration number. | Page 4 |
| Eligibility criteria | 6 | Specify characteristics of the sources of evidence used as eligibility criteria (e.g., years considered, language, and publication status) and provide a rationale. | Page 10 |
| Information sources* | 7 | Describe all information sources in the search (e.g., databases with dates of coverage and contact with authors to identify additional sources), as well as the date the most recent search was executed. | Page 11,12-16 |
| Search | 8 | Present the full electronic search strategy for at least 1 database, including any limits used, such that it could be repeated. | Page 6-7 |
| Selection of sources of evidence† | 9 | State the process for selecting sources of evidence (i.e., screening and eligibility) included in the scoping review. | Page 8-9 |
| Data charting process‡ | 10 | Describe the methods of charting data from the included sources of evidence (e.g., calibrated forms or forms that have been tested by the team before their use, and whether data charting was done independently or in duplicate) and any processes for obtaining and  confirming data from investigators. | Page 5-6 |
| Data items | 11 | List and define all variables for which data were sought and any assumptions and simplifications made. | Page 7, 8-9 |
| Critical appraisal of individual sources of evidence | 12 | If done, provide a rationale for conducting a critical appraisal of included sources of evidence; describe the methods used and how this information was used in any data synthesis (if appropriate). | Not compulsory for scoping reviews |
| Synthesis of results | 13 | Describe the methods of handling and summarizing the data that were charted. | Page 10 |

| **SECTION** | **ITEM** | **PRISMA-ScR CHECKLIST ITEM** | **REPORTED ON PAGE #** |
| --- | --- | --- | --- |
| **RESULTS** | | | |
| Selection of sources of evidence | 14 | Give numbers of sources of evidence screened, assessed for eligibility, and included in the review, with reasons for exclusions at each stage, ideally using a flow diagram. | Page 8-9 |
| Characteristics of sources of evidence | 15 | For each source of evidence, present characteristics for which data were charted and provide the citations. | Page 17-22 |
| Critical appraisal within sources of evidence | 16 | If done, present data on critical appraisal of included sources of evidence (see item 12). | Not applicable |
| Results of  individual sources of evidence | 17 | For each included source of evidence, present the  relevant data that were charted that relate to the review questions and objectives. | Not applicable |
| Synthesis of results | 18 | Summarize and/or present the charting results as they relate to the review questions and objectives. | 13,14,15 - 23 |
| **DISCUSSION** | | | |
| Summary of evidence | 19 | Summarize the main results (including an overview of concepts, themes, and types of evidence available), link to the review questions and objectives, and consider the relevance to key groups. | Pages 13,14-23  Pages 28,29-30 |
| Limitations | 20 | Discuss the limitations of the scoping review process. | Page 31 |
| Conclusions | 21 | Provide a general interpretation of the results with respect to the review questions and objectives, as well as potential implications and/or next steps. | Page 32 |
| **FUNDING** | | | |
| Funding | 22 | Describe sources of funding for the included sources of evidence, as well as sources of funding for the scoping review. Describe the role of the funders of the scoping review. | Page 33 |

JBI = Joanna Briggs Institute; PRISMA-ScR = Preferred Reporting Items for Systematic reviews and Meta-Analyses extension for Scoping Reviews.

* Where *sources of evidence* (see second footnote) are compiled from, such as bibliographic databases, social media platforms, and Web sites.

† A more inclusive/heterogeneous term used to account for the different types of evidence or data sources (e.g., quantitative and/or qualitative research, expert opinion, and policy documents) that may be eligible in a scoping review as opposed to only studies. This is not to be confused with *information sources* (see first footnote).

‡ The frameworks by Arksey and O’Malley (6) and Levac and colleagues (7) and the JBI guidance (4, 5) refer to the process of data extraction in a scoping review as data charting*.*

§ The process of systematically examining research evidence to assess its validity, results, and relevance before using it to inform a decision. This term is used for items 12 and 19 instead of "risk of bias" (which is more applicable to systematic reviews of interventions) to include and acknowledge the various sources of evidence that may be used in a scoping review (e.g., quantitative and/or qualitative research, expert opinion, and policy document).

*Source:* Tricco AC, Lillie E, Zarin W, O'Brien KK, Colquhoun H, Levac D, et al. PRISMA Extension for Scoping Reviews (PRISMAScR): Checklist and Explanation. Ann Intern Med. 2018; 169:467–473. [doi: 10.7326/M18-0850.](http://annals.org/aim/fullarticle/2700389/prisma-extension-scoping-reviews-prisma-scr-checklist-explanation)

SI Appendix 2: Data Extraction Template

| **Study, name of first author and year** | **Study country/ region** | **Aim(s) of the study** | **Study Method / design** | **Type of Data Collection / Approach** | **Population size (number of women)** | **Prevalence rates of CS per study region** | **Key findings on of CS barriers by the primary author(s)** | **Key findings on of CS facilitators by the primary author(s)** |
| --- | --- | --- | --- | --- | --- | --- | --- | --- |
|  |  |  |  |  |  |  |  |  |

Source: Peters et al. (2020a)

SI Appendix 3: Searches planned for each database and rationale 4^th^ -5th April 2024

| Database | Rational | Search string |
| --- | --- | --- |
| CINAHL  (Ebsco) | CINAHL includes journals on nursing, midwifery and the other health professions. | 1.Caesarean section* OR Cesarean section* OR C-Section*  2.Prevalence* OR Rate* OR Uptake* OR Barrier* OR Facilitator* OR Factor* OR Determinant*  3.(Prevalence* OR Rate* OR Uptake* OR Barrier* OR Facilitator* OR Factor* OR Determinant*) AND (S1 AND S2)  4.Ghana* OR West Africa*  5.(Ghana* OR West Africa*) AND (S3 AND S4) |
| Ovid Embase | Widely includes more international journals than Medline on biomedical and applied health sciences | 1.''caesarean section*''.mp.  2.''cesarean section*''.mp.  3.''c-section*''.mp.  4.1 or 2 or 3  5.prevalence*.mp.  6.Rate*.mp.  7.Epidemiology*.mp.  8.Uptake*.mp.  9.Barrier*.mp.  10.facilitator*.mp.  11.factor*.mp.  12.determinant*.mp.  13.5 or 6 or 7 or 8 or 9 or 10 or 11 or 12  14.4 and 13  15.Ghana*.mp.  16.West Africa*.mp.  17.15 or 16  18.14 and 17  19.limit 18 to (english language and humans) |
| (Ovid) PsycINFO | Largely includes international journals on midwifery, nursing, clinical medicine among others. | 1.''caesarean section*''.mp.  2.''cesarean section*''.mp.  3.''c-section*''.mp.  4.1 or 2 or 3  5.prevalence*.mp.  6.Rate*.mp.  7.Epidemiology*.mp.  8.Uptake*.mp.  9.Barrier*.mp.  10.facilitator*.mp.  11.factor*.mp.  12.determinant*.mp.  13.5 or 6 or 7 or 8 or 9 or 10 or 11 or 12  14.4 and 13  15.Ghana*.mp.  16.West Africa*.mp.  17.15 or 16  18.14 and 17  19.limit 18 to (english language and humans) |
| Web of Science (Clarivate) | Multidisciplinary in nature as it cuts across the social sciences, sciences and humanities etc. | 1.Topic: Caesarean section OR ceasarean section OR c-section  2. Topic: Prevalence OR rate  3. Topic: Uptake OR utilization OR access  4. Topic: Facilitator*OR barrier* OR factor* OR influence* OR decision making OR peer influence  5. Topic: Ghana OR west* Africa OR western*Africa OR sub-Saharan Africa  6.#1 AND #2 AND #3 AND #4 AND #5 |

SI Appendix 4: Summary of the code book adopted

| SI Appendix 4: Review’s Code Book using the TDF | | |
| --- | --- | --- |
| **No** | **TDF Domain (brief description)** | **Description/Coding Rule (as applicable to this review)**  HCPs’ / patient (mothers’) comments related to: |
| 1. | **Environmental Context and Resources**  Any circumstance of a person’s situation or environment that discourages or encourages the development of skills and abilities, independence, social competence, and adaptive behaviour.  **Includes:**  Environmental stressors, resources/material resources, organisational culture/climate, salient events/critical incidents, person x environment interaction, barriers and facilitators. | • The logistical factors influencing the decision to have or perform a CS including the availability of healthcare facilities, the time available in emergency settings, and the ability to pay cash or with insurance coverage.  • Institutional policies and guidelines and how they influence the prevalence of CS  • How cultural or societal factors affect access to information or resources related to childbirth options |
| 2. | **Social Influences**  Those interpersonal processes that can cause individuals to change their thoughts, feelings, or behaviours  **Include:**  Social pressure: the exertion of influence on a person or group by another person or group.  Social norms: Socially determined consensual standards that indicate what behaviours are considered proper in the context.  Power: The capacity to influence others, even when they try to resist this influence. | •How family members' opinions or experiences influence mothers' decision-making regarding CS.  •Healthcare professionals' recommendations or preferences that affect mothers' choices.  •Societal norms and cultural beliefs that shape attitudes towards CS. |
| 3. | **Belief About Consequences**  Acceptance of the truth, reality or validity about outcomes of a behaviour in a given situation.  **Includes:**  Beliefs: The thing believed; the proposition or set of propositions held true  Outcome expectancies: Cognitive, emotional, behavioural, and affective outcomes that are assumed to be associated with future or intended behaviour. These assumed outcomes can either promote or inhibit future behaviours. | • The perceived benefits of choosing a CS for mothers and benefits to the healthcare professionals.  • The perceived risks or negative consequences associated with CS  • Weighing the short-term benefits such as convenience against the long-term risks such as complications in subsequent pregnancies now and in future pregnancies. |
| 4. | **Emotion**  A complex reaction pattern, involving experiential, behavioural and physiological elements, by which the individual attempts to deal with a personally significant matter or event.  **Includes:**  Fear: An intense emotion aroused by the detection of imminent threat, involving an immediate alarm reaction that mobilizes the organism by triggering a set of physiological changes.  Anxiety: A mood state characterized by apprehension and somatic symptoms of tension in which an individual anticipates impending danger, catastrophe or misfortune. | •The emotions mothers experience when considering a CS such as anxiety, fear, and relief.  • Healthcare professionals' emotions concerning culture, societal norms, litigation, profit gains and the impact on their views on CS. |
| 5. | **Knowledge**  Knowledge (including knowledge of condition/scientific rationale): An awareness of the existence of something.  **Includes:**  Procedural knowledge: knowing how to do something.  Knowledge of task environment: Knowledge of the social and material context in which a task is undertaken. | • Women’s knowledge about CS and the implication of having a CS e.g. sides effects, recovery time, pain management.  • The knowledge mothers and healthcare professionals have about the indications for a CS. Knowing the impact of HCP’s recommendations for mode of birth behaviour 1 and women’s decision to undergo a CS or vaginal birth behaviour 2. |
| 6. | **Beliefs About Capabilities**  Acceptance of the truth, reality, or validity about an ability, talent or facility that a person can put to constructive use  **Includes:**  Self-confidence: Self-assurance or trust in one’s own abilities, capabilities and judgement  Perceived competence: An individual’s belief in her or her ability to learn and execute skills | •The confidence that HCPs have in themselves or other HCPs to manage labour, perform surgical procedures, handle potential complications, and achieve desired birth outcomes.  • Level of mothers’ confidence to make the decision on their own about having a CS. |
| 7. | **Memory and attention**  The ability to retain information, focus selectively on aspects of the environment and choose between two or more alternatives.  **Includes:**  Memory: The ability to retain information or a representation of a past experience, based on the mental processes of learning or encoding retention across some interval of time, and retrieval or reactivation of the memory; specific information of a specific task.  Attention: A state of awareness in which the senses are focussed selectively on aspects of the environment and the central nervous system is in a state of readiness to respond to stimuli. | • Mothers memories of past experiences of planning or actual childbirth mode and those of friends and family or information she has retained about childbirth.  • Positive or negative experiences or information learned relating to vaginal deliveries or CS by HCPs –and the perceived influence their preferences and perceptions of risk and benefit associated with each option. |
| 8. | **Skills**  An ability or proficiency acquired through practice.  **Includes:**  Skills: An ability or proficiency acquired through training and/or practice.  Competence: One’s repertoire of skills, and ability especially as it is applied to a task or set of tasks  Practice: Repetition of an act, behaviour, or series of activities, often to improve performance or acquire a skill.  Ability: Competence or capacity to perform a physical or mental act. Ability may be either unlearned or acquired by education and practice | •The surgical skills of HCPs and its impact on their decision to recommend CS.  •The self-management, recovery and care skills in wound care, pain management and its impact on their decision to have a CS/vaginal birth. |
| 9. | **Optimism**  The confidence that things will happen for the best or that desired goals will be attained.  **Include:**  Optimism: The attitude that outcomes will be positive, and that people’s wishes or aims will be ultimately fulfilled | • Weighting the short-term benefits such as convenience against the long-term risks such as complications in now and subsequent pregnancies.  • The belief women have that CS will provide the best outcome of childbirth.  •The belief women have that vaginal birth offer a safer or more predictable option for childbirth. |
| 10. | **Reinforcement**  Increasing the probability of a response by arranging a dependent relationship, or contingency, between the response and a given stimulus.  **Includes:**  Rewards (proximal/distal, valued/ not valued, probable/improbable): Return or recompense made to, or received by a person contingent on some performance. | •The impact positive reinforcement impacts on HCP recommendations to women regarding mode of birth in recognition from colleagues, and perceived success in practice.  •The impact of negative reinforcement on HCP recommendations to women regarding mode of birth such as fearing criticism, blame or legal consequences if complications arise. |
| 11. | **Behaviour regulation**  Anything aimed at managing or changing objectively measured actions.  **Include:**  Self-monitoring: A method used in behavioural management in which individuals keep a record of their behaviour, especially in connection with efforts to changes or regulate the self; a personality trait reflecting an ability to modify one’s behaviour in response to a situation.  Action planning: The action or process of forming a plan regarding a thing to be done or a deed. | •Factors influencing mothers' ability to adhere to their chosen mode of delivery such as birth plans and their coping strategies.  •Healthcare professionals regulating their behaviours in providing information, guidance, or care related to CS. |
| 12. | **Intentions**  A conscious decision to perform a behaviour or a resolve to act in a certain way.  **Includes:**  Stability of intentions: ability of one’s resolved to remain in spite of disturbing influences.  The inclination of whether they will be influenced to do something or not. | • The intentions or motivations driving mothers' decisions to opt for a CS.  • Healthcare professionals' intentions aligning with evidence-based practice guidelines or personal beliefs about childbirth. |
| 13. | **Goals**  Mental representations of outcomes or end states that an individual wants to achieve.  **Includes:**  Goal priority: Order of importance or urgency of end state toward which one is striving to achieve.  Goals (autonomous/controlled): The end state toward which one is striving: the purpose of an activity or endeavour. It can be identified by observing that a person ceases or changes their behaviour upon attaining this state; proficiency in a task to be achieved within a set period of time. | •The desired outcomes for mothers and healthcare professionals in choosing a mode of delivery for women.  •Goals that align with or conflict with each other such as maternal preferences verses healthcare professionals' clinical judgment. |
| 14. | **Social/Professional Role and Identity**  A coherent set of behaviours and displayed personal qualities of an individual in a social or work setting.  **Includes:**  Professional identity: The characteristics by which an individual is recognised relating to, connected with or befitting a particular profession. | • Healthcare professionals' identities and their roles influencing their approach to recommending or performing CS.  • Mothers' perceptions and their roles as mothers that influence their decision-making regarding childbirth options. |

*Source:* Adapted from: Atkins et al., (2017)

SI Appendix 5: Evidence of the TDF specific domains coded by studies

| **No** | **TDF Domains Coded** | **Studies Coded** | **Example Quotes** |
| --- | --- | --- | --- |
| 1. | Environmental Context and Resources | Rishworth et al., (2016), Bam et al., (2021), Asah-Opoku et al., (2023), Apanga et al., (2018), Gandau et al., (2019), Islam et al., (2022), Banchani et al., (2020). | … ‘’The roads here are very bad and there’s no transport, so we usually struggle to reach a health centre. By the **time we get** there, it’s always late and pregnancy gets complicated along the way, so you get a C-section’’. Mother participate during focus group discussion. Rishworth et al., (2016). |
| 2. | Social Influences | Asah-Opoku et al., (2023), Darteh (2020), Rishworth et al., (2016), Gandau et al., (2019), Konlan et al., (2019). | “…The best way to deliver is like the Hebrew women (vaginally), that’s what is in the Bible”. Mother participant in focus group in Asah-Opoku et al., (2023).  … “They [the women] say CS is not the ideal plan of God’’. House officer interview in Asah-Opoku et al., (2023). |
| 3. | Beliefs About Consequences | Rishworth et al., (2016), Asah-Opoku et al., (2023), Bam et al., (2021), Gandau et al., (2019). | … ‘’The C-section is much more difficult to do than normal delivery. There are complications that develop later in labour so if you don’t take care of it, the person may die. So, if they’re performing a C-section, the doctors are aware and think it’s right for the woman’’ Mother participant during in-depth interview. Rishworth et al., (2016).  … “CS was considered by women to be dangerous, ‘unnatural’ based on religious beliefs, and as a procedure that ‘takes away their strength’. The perceived diminished strength causes others in the community to perceive mothers as lazy, weak, and fragile”. Study findings, Asah-Opoku et al., (2023).  …” I had heard there was the possibility of losing one’s life”. Mother participant during interview”. Asah-Opoku et al., (2023). |
| 4. | Emotion | Asah-Opoku et al., (2023), Konlan et al., (2019), Adu-Bonsaffoh et al., (2022), Rishworth et al., (2016). | …’’ The fear of mothers for CS and their perception of CS as being unnatural due to religious beliefs were also recognized by health care professionals during interviews. Study findings, Asah-Opoku et al., (2023). |
| 5. | Knowledge | Rishworth et al., (2016), Asah-Opoku et al (2023), Gandau et al., (2019). | “…Yes! Abruption Placenta – the placenta was torn”, Mother participate during interviews using questionnaires in Asah-Opoku et al., (2023).  ‘’… I wish they could have told me much more about the risks before making me do it’’ Mother participant during a focus group discussion in Asah Opoku et al., (2023). |
| 6. | Beliefs About Capabilities | Rishworth et al., (2016), Asah-Opoku et al., (2023), Bam et al., (2021). | …’’According to the health care professionals …’’mothers usually have a low self-esteem to make decisions on their own’’. Study findings by Asah-Opoku et al., (2023), |
| 7. | Memory, attention, and decision processes. | Bam et al., (2021), Rishworth et al., (2016). |  |
